# Remembering change: Interdependence between change awareness and meaningful connection in achieving proactive facilitation

**DOI:** 10.3758/s13421-024-01651-3

**Published:** 2024-10-25

**Authors:** Paula T. Hertel, Christopher N. Wahlheim, Grant M. Kramer, Faith L. Padgett

**Affiliations:** 1 Psychology Department, Trinity University, 1 Trinity Place, San Antonio, TX 78212 USA; 2 Psychology Department, University of North Carolina at Greensboro, Greensboro, NC 27402 USA

**Keywords:** Proactive facilitation, Proactive interference, Change detection, Integration, Meaning

## Abstract

Two experiments investigated proactive facilitation (PF) or proactive interference (PI) in the recall of recently learned targets, under conditions of assessing the detection and recollection of target changes across two learning phases (with A-B/A-D word pairs). Some changes established meaningful connections across the phases; others did not. Task instructions on the subsequent cued-recall test (Experiment 1) or during Phase 2 study (Experiment 2) guided participants (university students) to monitor and report the changes. Accuracy in cued recall conditionalized on measures of change awareness replicated previous findings in establishing conditions for PF and PI. However, PF was much reduced for unconnected materials. Moreover, when change recollection failed, PI occurred even under conditions of meaningful connections (Experiment 1). Discussion emphasizes this interdependence of meaningfulness of connections and change awareness in influencing whether and how memory for earlier events affects memory for more recent ones.

Across decades, experimental psychology has established the power of initial learning to influence memory for subsequent changes. Early experiments documented the role of initial learning of cue–target pairs in impairing recall of later learned associates to the same cues—a phenomenon known as proactive interference (for reviews, see Anderson & Neely, 1996; Postman & Underwood, 1973). Later research on Pavlovian conditioning revealed that second-learned modifications like extinction and counterconditioning can crumble under the greater generalizability and functional durability of the first-learned contingency (Bouton, 1993). These are disturbing truths for all situations in which memory for change is crucial. Fortunately, however, these early investigations and more recent ones also addressed conditions under which the power of the first learned can be tamed and even harnessed to facilitate memory for change. Our experiments focus on the conditions for such proactive facilitation by calling attention to the importance of meaningful connections between the earlier and later events, especially when changes across events have been well detected.

Inspired by the classic interference experiments, more recent research has examined the role of change awareness in later recall of the updated targets (for a review, see Wahlheim et al., 2021). Like the classic work, the more recent experiments often used A-B/A-D paired-associate learning tasks to show that exposure to A-B pairs either proactively interfered with or facilitated cued recall of the later-studied D targets. The critical insight from this recent work is that awareness of changes—at the time that the changes occur and during subsequent attempts to recall them—is critical for observing proactive facilitation. This awareness can be brought under control through instructions and task design; for example, asking participants to retrieve the corresponding B target while studying each A-D pair led to better memory for the changed (D) targets (and heightened awareness that they had changed) compared to control pairs (Jacoby et al., 2015). This awareness may also vary with individual differences in the ability to control attention. This was shown when people who better sustained their attention while studying changes also better remembered changed (D) targets and the fact that they changed (Garlitch & Wahlheim, 2020). Change awareness is therefore important, but so are meaningful connections between the competing targets.

Other lines of research with word pairs have revealed evidence of facilitation through varying the strength of semantic associations among words, both within and between pairs (Bennion et al., 2024; Wahlheim, 2014). A possible explanation of this category of facilitation effects points to the likelihood that they reflect undocumented change awareness, cued by the preexperimental, inherent semantic relations. In considering other examples of memory aided by meaningful connections to other sources (and possibly also by change awareness), we note that later events can retroactively enhance recall of earlier memories when cues and targets are more strongly associated, both within and between pairs (Antony et al., 2022; Barnes & Underwood, 1959). Thus, both the awareness of change and the degree of pre-established meaning among episodes can override typical sources of interference and even convert them to advantages. This report is an important first step to document their mutual dependence. However, our efforts regarding proactive effects are clearly compatible with other accounts of conditions for obtaining facilitation and interference, notably those addressing retrieval-practice effects (see Anderson & McCullough, 1999; Chan, 2009; see this General Discussion).

We designed the present experiments to discover whether the proactive facilitation associated with awareness of changes, both when changes are noticed and when later recollected, takes advantage of meaningful connections between the first- and second-learned pairs. The importance of this claim is illustrated by research in two applied domains: remembering corrections of misinformation and corrections of ruminative thoughts.

In the first case, misinformation contained in narratives (such as news reports) continues to influence measures of memory and judgment, even when readers have read subsequent corrections and even when they later remember that corrections had occurred (for an early review, see Lewandowsky et al., 2012). This continued-influence effect aptly illustrates a common occurrence of proactive interference in everyday experiences, such as the reading of headlines. Yet reminders or other methods designed to increase awareness of the relation between the erroneous headline and its later correction actually promote proactive facilitation in memory for the correction, especially when participants remember at test that corrections had earlier occurred (e.g., Kemp et al., 2022; Wahlheim et al., 2020). The prominence of such awareness-associated proactive facilitation, similar to evidence from word-pair experiments, suggests that facilitation is boosted by the inherent meaning in the connections between the original and corrected headlines. The benefits of meaningful connections may also contribute to retroactive misinformation effects (for a review, see Loftus & Klemfuss, 2023) in which memory for observed event is impaired by reading misinformation in later narratives. Memory for original events can be retroactively facilitated when participants notice and later recollect the discrepancies between the event and the misleading narrative (Putnam et al., 2017). In applications like these, moreover, awareness seems likely encouraged or even potentiated by the inherently meaningful associations between the otherwise competing sources. Indeed, the very notion of correction implies connected meaning.

The benefits of proactive facilitation through meaningful connections might also be important in the domain of clinical psychology. Repetitive or ruminative thinking about past problematic events—a characteristic of anxious and depressed states—ensures the continuous influence of those memories (e.g., Hertel et al., 2020), to the detriment of interim attempts to train more benign interpretations (see Everaert et al., 2018). And there is even some evidence that the sticky memories embedded in rumination interfere with therapeutic interventions (see Watkins & Roberts, 2020). Unfortunately, the “corrections” established by the interventions may not easily come to mind outside the context of therapy, where the first-learned thoughts reemerge through renewal. Methods for preventing relapse by counteracting this type of proactive interference have been offered by analogy to conditioning paradigms (Bouton, 2000). More recently, we used the A-B/A-D paradigm to model proactive facilitation in remembering aspects of therapeutic interventions (Hertel et al., 2023). Participants in those experiments were students who clearly did or did not describe themselves as ruminators. They first-learned negative word pairs, then learned related but benignly corrective targets associated with the same cues (e.g., *body–shame* then *body–comfort*), and finally tried to recall the benign targets while monitoring memory for changes. As anticipated, the recall performance of both the ruminators and the other students revealed proactive interference when change recollection failed and proactive facilitation when it succeeded. But the important finding for the current purpose was that the ruminators showed significantly greater proactive facilitation than did the others; pairs from the two learning phases were meaningfully connected, but the connections were likely more meaningful to students with the habit to ruminate about negative life events.

More generally, we propose that meaningful connections between initial and changed events facilitate memory for the changed events, under conditions of change detection and recollection. Similar to the previous experiments on rumination but lacking obvious emotional meaning, the “events” in the current experiments were target words that were separately learned with a shared cue (e.g., porch–*light*, porch–*moth*) and shared contextually established meaning (e.g., porch and moth are related in the context of the light). We designed the experiments to reveal the importance of meaningful connection when change is explicitly interrogated, either during the recall test (change recollection; Experiment 1) or during the learning of the changed targets (change detection; Experiment 2). Our goals were first to document the importance of connection to proactive facilitation through change recollection and, second, to examine whether such connection-assisted effects can obtain when change is explicitly evaluated during its encounter. That procedure can be more easily exploited in applied domains such as therapy or fact-checking the claims of politicians.

## Experiment 1

### Transparency

This experiment was preregistered on the Open Science Framework, with a predetermined sample size (search for zm9aq). The materials, instructions, data, and code can be found online (search for xr2zn). 

### Method

#### Participants

As indicated in the preregistration: G*Power (Version 3.1.9; Faul et al., 2007) suggested the target sample size of 36 when we set alpha at 0.05 and power to detect an effect size of *d* = 0.5^1^ at 0.90 in a dependent-samples *t* test (for the critical comparisons of conditionalized recall of changed targets to the recall from control pairs). Our stopping rule was to test 12 qualified participants in each of three versions of the experimental tasks (versions established by counterbalancing materials with experimental conditions; see description below). “Qualified” participants followed instructions, passed on fewer than half of the trials, and recalled more than one Phase 2 target. These inclusion criteria indicated some minimal effort to comply. To reach that point, experimenters recruited 45 students from the pool of students enrolled in the introductory course in psychology at Trinity University. We excluded data from nine students who did not meet our inclusion criteria.^2^ Participants were randomly assigned to task versions with the constraint of reaching 12 in each. The final sample included 36 students (*M*_age_ = 18.8 years) who identified as women (61%), men (36%), and nonbinary (3%); White/Caucasian (50%), Hispanic/Latinx (30%), East or Southeast Asian (17%), and Black/African American (3%).

#### Materials and design

The experimental session consisted of two learning phases and a test phase. To vary connections across the two learning phases, we constructed 36 word-quadruplets, each containing one cue (A), two targets for Phase 1 (B or C), and one target for Phase 2 (D). D targets were the focus of subsequent cued recall. We describe targets from A-B/A-D pairs as being “connected,” and targets from A-C/A-D pairs as being “unconnected,” although clearly these two categories represent variation on a continuum. The relational difference between the two conditions was confirmed by pilot data from a rating task in which students judged the conceptual relation of D targets to either A-B or A-C pairs on a 7-pt scale (1 = *unrelated* to 7 = *strongly related*; *M* = 5.4 for connected pairs vs. 2.4 for unconnected), *t*(35) = 17.3, *p* < 0.001, *d* = 2.88. A-B pairs and D targets (e.g., *porch–light* and *moth*) were more strongly related than were A-C pairs and D targets (e.g., *porch–swing* and *moth*). Table 1 provides additional examples from each of these conditions. When imagination allowed, we constructed the quadruplets to reflect coherent scenarios in the connected condition (e.g., in Table 1, there is a view from the corner office; the ladder by the tree leads to the tree house; the horse won a prize in the race). The B terms typically provide a context for the relation between A and D. Notably, however, the free-association norms (De Deyne et al., 2019) report lower associative frequencies for B targets than for C targets in response to A cues (*M* = 0.03 vs. 0.09, respectively).

**Table 1:** Examples of word pairs

Condition	Phase 1	Phase 2 (A-D)	Test (D)
Connected	**corner-office**	**corner-view**	**corner-?**
(A-B)	*tree–house*	*tree–ladder*	*tree–?*
	horse–race	horse–prize	horse–?
Unconnnected	corner–store	corner–view	corner–?
(A-C)	**tree–leaf**	**tree–ladder**	**tree–?**
	*horse–carriage*	*horse–prize*	*horse–?*
Control	–	*corner–view*	*corner–?*
	–	tree–ladder	tree–?
–	–	**horse–prize**	**horse–?**

As an illustration of counterbalancing, font type links the pairs shown to three different participants

For counterbalancing, we sorted the quadruplets into three sets of 12, one to be assigned to the connected condition, another to the unconnected condition, and the third to the control condition, in which corresponding pairs were not presented in Phase 1. Set membership was determined by simultaneously balancing the sets on word frequency and concreteness of the targets (Brysbaert et al., 2014), their emotional valence (Warriner et al., 2013), and on our pilot ratings for the meaningfulness of the connections. Then to counterbalance materials with conditions, sets rotated across the three conditions.

##### Filler trials

Our primary materials constituted 24 trials in which the target changed (12 connected and 12 unconnected) and 12 control trials. To provide the needed additional materials for the full design used in A-B/AD experiments that include measures of change awareness, we also presented 36 filler pairs from previous PF experiments (e.g., *walnut–squirrel, soup–sandwich*; Hertel et al., 2023; Wahlheim, 2015). Of the 36 fillers, 24 were assigned to serve as repeated pairs across the two learning phases, and 12 were assigned to control trials. These assignments were fixed, because as filler trials, their data were not examined. Yet it was important that participants experience an equal number of repeated, changed, and new (control) targets to prevent biased responding when participants judge whether target responses changed, even though tests of proactive facilitation require only changed and control trials.

##### Presentation orders and buffers

In each phase, we assigned the pairs to 12 fixed blocks and randomized presentation order within the blocks. In Phase 1, each block contained an A-B pair (meaningfully connected to the D target in Phase 2), an A-C pair (unconnected), and two filler pairs to be repeated in Phase 2 to total 48 trials. Those cue-to-block assignments were maintained in the Phase 2 order, but D targets replaced B or C targets. In addition, each Phase 2 block contained one control pair and one filler control pair. Thus, all 72 A-D pairs (including fillers) appeared in Phase 2. Moreover, those 72 cues appeared once each on the test, with the same block assignment as in Phase 2. Three buffer pairs also appeared at the beginning and three at the end of Phase 1. Then we repeated one of the beginning buffers in Phase 2, changed the target in the other two, and added a new pair; the ending buffers in Phase 2 contained one repeated, one changed, and one new pair.

#### Procedure

Instructions and trials were implemented with Qualtrics software (Qualtrics, Provo, UT) on a desktop computer. An experimenter sat with the participant during instructions in each phase, worked through the first buffer trial, then sat behind a screen while the trials were in progress.

##### Learning phases (Phases 1 and 2)

On each trial in Phase 1, the cue–target pair appeared center screen for 5 s, and the participant read it aloud and constructed a mental image to represent its meaning. With the offset of the pair, the participant rated the difficulty in forming the image by using a 5-pt scale. This rating was self-paced. (These instructions replicated those from Hertel et al., 2023, which were designed to promote self-involvement with the materials.) Phase 2 began after a short break, with instructions to read each pair aloud and study it for a test that would evaluate their recall of the target when the cue appeared. Participants were also asked to notice any connections between these pairs and the pairs in the previous phase. (No overt response was required.) We told them that some pairs would be the same as previously studied, other pairs would have the same cues but different targets, and still others would be entirely new. Pairs were again presented for 5 s each, without a subsequent rating.

##### Test

As in previous tests of memory benefits associated with change recollection, each test trial consisted of two or three steps: Upon presentation of the cue, the first step was to recall the target from Phase 2, guessing if necessary, and passing as a last resort. The second step was to decide whether that target was repeated across the learning trials, changed from the target studied in Phase 1, or never seen earlier (new); the options of repeated, changed, and new appeared next to radio buttons that participants clicked. In the third step—required whenever “changed” was clicked—a text box appeared with a request to recall the corresponding Phase 1 target. The experimenters used the three test buffers to check comprehension and correct misunderstandings before starting the actual test. The test was self-paced, and all 72 cues appeared. Following the test, participants filled out questionnaires (unrelated to our results) and provided information about gender, age, and race or ethnicity, prior to debriefing.

### Statistical methods

We conducted all analyses from both experiments by using R software (R Core Team, 2021) and examined the effects of interest with logistic mixed-effects models using the glmer function from lme4 (Bates et al., 2015). The models included a fixed effect of the prior learning conditions and random intercept effects of subjects and items. We performed Wald’s χ^2^ hypothesis tests with the Anova function of the car package (Fox & Weisberg, 2019) and pairwise comparisons with the Tukey method using the emmeans function from emmeans (Lenth, 2021). The model specifications are available on the OSF (https://osf.io/3zc7d/). The significance level was α = 0.05. When possible, we report odds ratio (*OR*) effect sizes.

### Results and discussion

#### Evidence for proactive facilitation (PF) or proactive interference (PI)

**Fig. 1:**
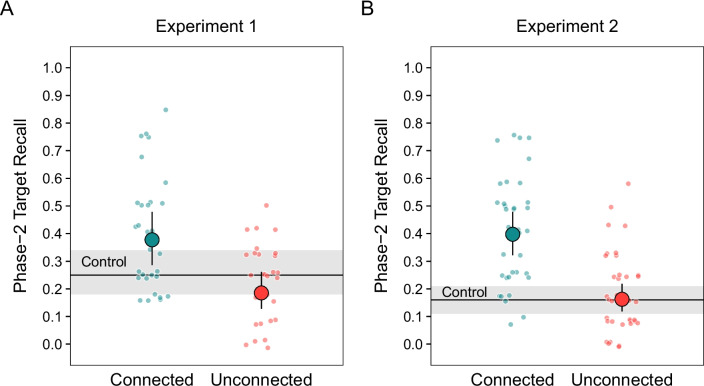
Probabilities of Phase 2 target recall. Phase 2 target recall in Experiment 1 (**A**) and Experiment 2 (**B**). The larger points above and horizontal bars are estimated probabilities derived from mixed-effect models. The error bars and shaded regions are 95% confidence intervals. The smaller points are individual participant probabilities

Analysis of the proportion of Phase 2 targets recalled revealed significant differences among the estimated marginal means, χ^2^(2) = 30.26, *p* < 0.001 (see Fig. 1A). The comparison of the connected condition with the control condition revealed PF, *z* ratio = 3.34,* p* < 0.01, *OR* = 1.78. In contrast, neither PF nor PI were found in the unconnected condition, *z* ratio = 2.17, *p* = 0.08, *OR* = 0.67.

#### PF under conditions of change recollection

**Fig. 2:**
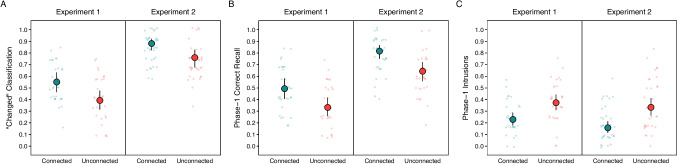
Probabilities of “changed” classification, Phase 1 correct recall, and Phase 1 intrusions. “Changed” classification (**A**), Phase 1 correct recall (**B**), and Phase 1 intrusions (**C**) in Experiments 1 and 2. The larger points above are estimated probabilities derived from mixed-effect models. The error bars are 95% confidence intervals. The smaller points are individual participant probabilities

Following the attempt to recall each Phase 2 target, participants judged whether it had been repeated or changed from the target learned in Phase 1 or belonged to a new pair in Phase 2. Figure 2A shows that they chose “changed” more often in the connected than unconnected condition, *z* ratio = 3.95, *p* < 0.001, *OR* = 1.89. (Participants thought that change had occurred on just 4% of control trials.) Moreover, connection facilitated Phase 1 target recall after change was indicated (see Fig. 2B), *z* ratio = 4.09, *p* < 0.001, *OR* = 1.96.

**Fig. 3:**
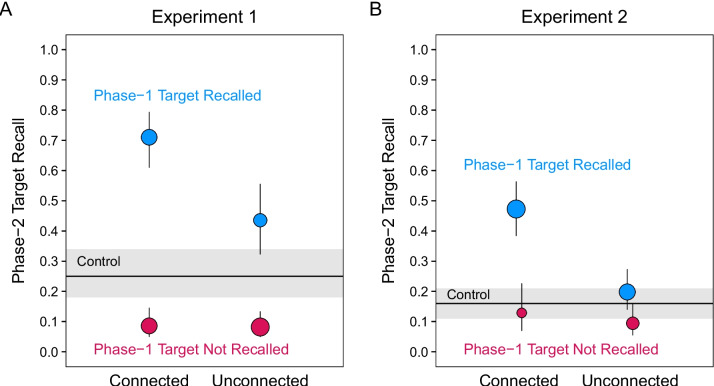
Probabilities of Phase 2 target recall conditioned on Phase 1 target recall. Conditional Phase 2 target recall in Experiment 1 (**A**) and Experiment 2 (**B**). Points represent estimated probabilities derived from mixed-effect models. The error bars are 95% confidence intervals

More centrally, if change recollection is associated with PF, participants should recall more of the Phase 2 targets if they also recall the corresponding Phase 1 target. In addition, if PI is more likely in the absence of change recollection, participants should recall fewer Phase 2 targets when they do not recall the corresponding Phase 1 target. Figure 3A displays these conditional probabilities, obtained from our data.

PF was obtained in both conditions when Phase 1 targets were recalled. When compared with the control condition, Phase 2 target recall was significantly higher in the connected condition, *z* ratio = 8.54, *p* < 0.001, *OR* = 7.02, and in the unconnected condition, *z* ratio = 3.28, *p* < 0.01, *OR* = 2.20. Of the two types, the benefit associated with Phase 1 recollection was greater in the connected than unconnected condition, *z* ratio = 4.27, *p* < 0.001, *OR* = 3.19. In contrast, PI was obtained in both conditions when Phase 1 targets were not recalled. When compared with the control condition, Phase 2 target recall was significantly lower in the connected condition, *z* ratio = 4.50, *p* < 0.001, *OR* = 0.27, and in the unconnected condition, *z* ratio = 5.42, *p* < 0.001, *OR* = 0.24. The two learning types showed comparable interference in the absence of recollection, *z* ratio = 0.31, *p* = 0.99, *OR* = 1.11. Notably, the absence of a connection effect in PI might be artifactual of the low levels of recall in all conditions.

#### Intrusions of Phase 1 targets

Figure 2C displays the probabilities of attempts to recall Phase 2 targets (D) that instead produced Phase 1 intrusions (B or C). Intrusions were more frequent in the unconnected than connected condition, *z* ratio = 4.11, *p* < 0.001, *OR* = 0.50. This outcome might occur for at least two reasons. First, the normative forward-association frequencies were higher in the unconnected condition. Second, the difficulty in recalling the Phase 2 target in the context of being asked not to pass should mean that the first-learned target would readily serve as an alternative response. That difficulty was arguably greater in the unconnected condition, due to the corresponding low level of meaningful connection between the targets. The finding of lower correct Phase 1 recall in the unconnected than connected condition (Fig. 2B) supports this assertion. Thus, intrusions were driven by strength of forward association to the cue, the requirement to avoid passing, or both.

## Experiment 2

### Transparency

Preregistration for Experiment 2 can be found by searching OSF for kwtu8. Materials, instructions, and data are stored at 3zc7d. Trinity University’s Institutional Review Board preapproved data collection.

### Method

The sample-size determination and the stopping rule were the same as in Experiment 1. A total of 39 students were recruited from a similar pool as in Experiment 1, and the data from three were excluded from analysis.^3^ The final sample of 36 students (*M*_*age*_ = 18.3 years) identified as women (72%), men (25%), and nonbinary (3%); White/Caucasian (53%), Hispanic/Latinx (28%), East or Southeast Asian (14%), and Black/African American (6%). For counterbalancing, they were randomly assigned, with the constraint of equal cell size, to the three versions of the task program.

All aspects of method were identical to Experiment 1, with three exceptions: First, the measure of awareness of change was deployed during the Phase 2 learning trials (i.e., change detection, instead of change recollection). Following the 5-s display of the A-D pair on each Phase 2 trial, participants indicated (by clicking radio buttons) whether the target was repeated, changed, or new. If they chose “changed,” a text box appeared with the instruction to recall the Phase 1 target (B or C). Second, participants were reminded at the beginning of the test of Phase 2 D targets that some had been repeated from Phase 1, some had been changed, and some pairs were new during Phase 2. In short, we reversed the location of each of these elements, compared to the procedure of Experiment 1. Third, after scorers noticed that some participants were recalling many Phase 1 targets on the test, we wondered if these were true intrusions or a deliberate strategy to avoid passing. We subsequently asked participants not to use such a strategy.

### Results and discussion

#### Evidence for proactive facilitation (PF) or proactive interference (PI)

As in Experiment 1, there was a significant effect of prior learning conditions, χ^2^(2) = 78.43, *p* < 0.001 (see Fig. 1B). PF in the connected condition clearly obtained, *z* ratio = 7.53, *p* < 0.001, *OR* = 3.54. Again, neither PF nor PI obtained in the unconnected condition, *z* ratio = 0.22, *p* = 0.97, *OR* = 1.04.

#### PF under conditions of change recollection

Because, in this experiment, participants made the change judgment immediately after studying each Phase 2 target, judgments were likely to be more accurate than in Experiment 1, yet a similar difference according to connection still obtained (see Fig. 2A; participants reported detecting changes on 7% of control trials.). “Changed” classification probabilities were significantly higher in the connected than unconnected condition, *z* ratio = 4.65, *p* < 0.001, *OR* = 2.33. And when participants detected change (see Fig. 2B), Phase 1 target recall in the connected condition was similarly superior, *z* ratio = 5.46, *p* < 0.001, *OR* = 2.45.

During instructions for the test of PF in this experiment, we included a general reminder about the previous changes in targets (as well as about the repetitions and novel pairs); the test presented only the cues. Nevertheless, having detected change and having recalled the first target during the learning of the second still strongly established PF when the pairs were connected (Fig. 3B), *z* ratio = 8.85, *p* < 0.001, *OR* = 4.75. However, in the unconnected condition, having previously recalled the Phase 1 target during Phase 2 did not significantly boost recall of the Phase 2 target beyond recall in the control condition, *z* ratio = 1.46, *p* = 0.59, *OR* = 1.34. Finally, when Phase 1 targets were not recalled earlier, Phase 2 target recall was not significantly different from the control condition in the connected condition, *z* ratio = 0.62, *p* = 0.97, *OR* = 0.81, or the unconnected condition, *z* ratio = 1.79, *p* = 0.38, *OR* = 0.60. This last result might suggest that failing to retrieve Phase 1 targets in Phase 2 undermined the potential for either PF or PI. However, recall of control targets was likely too low to allow evidence for PI or related effects involving meaningful connection.

#### Intrusions of Phase 1 targets

Erroneous recall of the first-learned targets (B or C) in place of the Phase 2 targets (D; Fig. 2C) was again more frequent in the unconnected than connected condition, *z* ratio = 5.82, *p* < 0.001, *OR* = 0.37. We discuss this finding briefly in Experiment 1 Results. In this experiment, however, the change in instructions approximately midway through data collection did reduce intrusion rates from 0.31 to 0.21, regardless of condition, but not significantly.^4^

## General discussion

The results of these two experiments supported our predictions and are best appreciated by considering the interdependence between change awareness and meaningful connection in facilitating memory for the changed targets. When initial and subsequently learned targets shared contextually established meaning, first-learned targets did not interfere with memory for the changed target, as long as the change had been detected or recollected. Instead, memory for the initial target proactively facilitated changed-target recall. This relation held true, regardless of when participants explicitly considered the connections between the two learning events, either while encountering the changed target or during the subsequent test trials. Fundamentally, these proactive effects replicate previous findings (see the review by Wahlheim et al., 2021). The novelty in the current results lies in the near restriction of replication to situations with contextually meaningful connections between the two learning events. Moreover, we found that shared meaning must be accompanied by change awareness (and memory for the first target) in order to facilitate memory for the changed target. That finding implies that previous evidence for facilitation by semantic relatedness might have relied on (undocumented) awareness of changes (cf. Bennion et al., 2024; Osgood, 1949). Next, we elucidate these interdependencies between change awareness and meaningful connection in both experiments.

Notably, the current results show that meaningful connections lose their positive influence if change is not detected; the connection must be noticed and remembered. To wit, facilitation was not obtained in the connected condition of either experiment without change detection or recollection. On the other hand, benefits associated with the awareness of change crumble when episodes lack meaningful connection. Change detection during exposure to the changed targets was not sufficient to produce facilitation in overall target recall in the unconnected condition (Experiment 2); such facilitation was obtained only when awareness measures were taken at the time of the test (in Experiment 1). Even then, however, facilitation under unconnected conditions was much less successful than under conditions of meaningful connection. In fact, this was the case even though intrusions from first-learned targets were more plentiful on the test (in both experiments). These targets came to mind but did not benefit the cued recall of corresponding Phase 2 targets as a result. Next, we place these outcomes in the context of the integration and interference accounts of proactive effects of memory.

### Frameworks for interpretation

The proposal that awareness of changes during learning can facilitate subsequent recall is inherent in integration accounts of episodic memory updating. The integration account most related to the present study, the memory-for-change framework (Wahlheim & Jacoby, 2013) proposes that recalling first-learned targets while studying second-learned targets can facilitate subsequent recall by establishing integrated representations that are later recollected at test. This account is based partly on the reminding accounts of temporal memory (e.g., Hintzman, 2010; Winograd & Soloway, 1985). The reminding accounts posit roles for event-cued retrieval of earlier memories in maintaining memory for relative event order. Features of currently perceived events can trigger retrieval of similar memories and thereby make both accessible on tests of studied material. As a class, these frameworks contrast with strict, classic interference theories proposing response competition between first and changed targets that impede new learning and later memory for the change (for a review, see Postman & Underwood, 1973). On the other hand, these frameworks (reminding accounts and memory for change) are compatible with mediation accounts, given the prominent role played by semantic relatedness (meaningful connection) in creating facilitation effects through mediated retrieval (e.g., Barnes & Underwood, 1959). However, mediation accounts lack an explicit role for change awareness and its representational consequences.

The question for the present experiments concerns the relation of meaningful connection to the mechanisms proposed by integration and interference accounts (generally) and the memory-for-change framework (specifically). Our results suggest that integration can be easily conceived from preexperimental meaning (e.g., our connected materials) or idiosyncratically forged out of ostensibly poorly connected elements (see Hunt, 2013), but the cognitive act of change detection, central to the memory-for-change framework, must happen first before either integrative process. In our unconnected condition, change was noticed less often. Importantly, when it was noticed, integration was hypothetically more difficult and often failed (although we only know that facilitation was poor, lacking an independent measure of ease of integration apart from the pilot ratings of relatedness). The memory-for-change framework also points to integration-aided retrieval processes; recall of either target on the test aids retrieval of the other one due to their prior integration. The advantage of meaning for mediation at retrieval (see Postman, 1971), by itself, does not seem likely in the absence of change detection and recollection, according to our results. Moreover, although evidence for interference obtained in conditional analyses, a strict interference account cannot explain the entire pattern of results because the negative effects of response competition occurred only in the absence of change awareness (i.e., detection or recollection).

More generally, numerous experiments have established facilitation when changes have been detected and later recollected and interference when detected changes are not later recollected (for review, see Wahlheim et al., 2021). Recollected changes at test almost exclusively reflect earlier-detected changes, while only a subset of changes detected during learning are later recollected at test (Wahlheim & Jacoby, 2013). Accordingly, differences in the magnitude of proactive facilitation observed across previously published experiments—and across conditions of connected meaning in the present experiments—may reflect the degree to which the measures of change awareness capture instances when participants can access integrated representations that include relative temporal order. The detection measure in Experiment 2, for example, should include fewer of those instances because its procedure could not reveal the detected changes that were not subsequently recollected. In short, an integration account can adequately address the current results. Indeed, the process called integration might otherwise be understood as detection of meaning inherent in or constructed from the connection between the two events when they are conceived in temporally close proximity.

Inherent meaning is easily observed in changes across everyday or routine episodes—the kinds of changes that can lead to difficulties like finding one’s keys—unless the nature of the change happens to be noted when it occurs. Findings from the event cognition literature are consistent with the proposal that meaningful connections and awareness of change together boost PF by promoting integration. In experiments on event memory updating (e.g., Wahlheim & Zacks, 2019; Wahlheim et al., 2022), participants watched movies of an actor performing actions in or around her home on 2 days in her life. Some of the actions included the same beginnings on both days with changed endings on the second day, such as when the actor approached the refrigerator retrieved water on the first day and milk on the second day. The subsequent test used brief action descriptions to cue recall of action endings and assessed memory for their change. Similar to the present findings, proactive facilitation obtained overall in recall of the more recent endings, and it was larger when recall was conditionalized on change awareness. Conversely, proactive interference obtained when participants noticed the change when it happened but did not recollect it when tested. Neuroimaging results have also implicated reinstatement of earlier-learned events in key regions of a contextual episodic memory network (i.e., the medial temporal lobe and posterior medial cortex) and other cortical regions in the noticing of changed actions that supports later memory for those actions (Stawarczyk et al., 2020). Collectively, these findings show that common experience (a version of inherent meaning) can provide the integrating glue across changes in the details of everyday actions, but the change must be both noticed and recollected to benefit memory for the most recent version.

The integration account is clearly a “representational” account of performance in facilitation and interference experiments. In contrast, a possible procedural account would question the necessity of the integration construct and place greater emphasis on the ease and frequency with which retrieval occurs. Even though unconnected targets in Phase 1 were slightly higher associates to their cues, connected targets might come to mind more readily during Phase 2 learning, because they were preexperimentally cued by the meaning of the new target (an outcome verified by the data in Experiment 2). Subsequently, on the test, the connected Phase 1 targets would also more readily come to mind, because they benefit from the previous retrieval episode (an advantage verified in Experiment 1 by recall of first-learned targets on trials in which changed targets were correctly classified as changed). And when these better connected first targets are retrieved they are more likely to recruit their corresponding changed targets by virtue of their preexperimental and mediational relations. In short, a role for the retrieval advantage established by preexperimental connections seems warranted. These experiments were not designed to distinguish between specific theoretical frameworks. Instead, we emphasize their common reliance on prior experience with meaning. Noticed during the change, meaningful connections become integrated episodic representations or such prior processing turns out to be transfer appropriate to retrieval efforts on memory tests (or both).

These considerations of retrieval processes remind us to point out an interesting and perhaps obvious correspondence between the conditions for obtaining proactive facilitation or interference on subsequent tests and the conditions under which retrieval practice produces either advantages or disadvantages in recalling unpracticed elements on subsequent tests (see Chan, 2009, for evidence and succinct discussion of the latter, as well as a review by Bäuml & Kliegl, 2017). Initially documented by Anderson and McCulloch (1999), a primary boundary condition for retrieval-induced forgetting is some degree of integration (or shared meaning) between the practiced and unpracticed events. Shared meaning can protect the unpracticed from later response competition from the practiced events, particularly when it is noticed during learning. As Chan (2009) has illustrated, the practiced items recalled on the test can serve as mediators in recalling the related unpracticed items and thereby produce retrieval-induced facilitation. Indeed, there is a clear correspondence between that analysis and our approach to proactive effects. Noticing meaningful connections, in general, protects from interference by the dominant (first or practiced) thing learned and facilitates memory for the “other thing” learned. (Postman and Underwood would not be greatly surprised.)

### Limitations

We turn now to a discussion of limitations and weaknesses. The first and most obvious aspect of method that invites attention is the unfortunate change in instruction midway through the collection of data in Experiment 2 (and a corresponding inability to replace the initial participants). However, the only effect of the instruction change that even approached significance on any measure was its main effect on the intrusion rate. Moreover, the patterns of mean across conditions of connection replicate across conditions of instruction (see the Appendix). A reasonable interpretation is therefore that the earlier participants indeed might have reported some Phase 1 responses to avoid passing on the test, but not because they had not tried to recall the correct targets. We can speculate that the intrusion data in both experiments, given the request not to pass, is an unstable representation of actual faulty recall. In other words, participants might have sometimes been aware of the source of the target that they reported inaccurately.

The second and perhaps less obvious issue with method concerns the difference in cue–target associative value within connected versus unconnected Phase 1 pairs (in both experiments). A-C (unconnected) forward associative values were higher that A-B values, and this difference might make the actual learning of D responses more difficult in the unconnected condition. However, we call attention to the trade-off between that possibility and the possibility that associative values for A-C as low as A-B (*M* = 0.03) would mean that C responses would have little chance of being available for change detection. (B responses had low normative probabilities of production but an advantage of being more easily cued by the A-D pair during Phase 2.) The relative size of each potentially confounding effect in this trade-off could not be determined. Moreover, the interference-during-learning confound is likely to have been subtle, given the low associative values of C responses (*M* = 0.09), compared to other differential retrieval effects documented in the literature on change detection. For example, Wahlheim and Jacoby (2013) manipulated the retrieval likelihood of first-learned responses through the number of their repetitions; more repetitions produced greater interference, however they also increased change detection, an outcome not obtained for our unconnected targets. Still, these issues are best avoided when it is possible to do so. The concept of meaningful connection across pairs seemed to require low levels of within-pair association. In short, construction was not as straightforward as we would prefer, but the associative difference is perhaps also not as powerful as it might seem.

Unsurprisingly, there are results from our experiments that cannot be easily understood and thereby also raise issues. The most obvious issues concern the low level of recall from control trials, particularly in Experiment 2. An absence of explicit awareness monitoring on the test inevitably produces lower levels of recall, compared with its inclusion (Experiment 1), if for no other reason than participants are unmotivated to carry out monitoring when they are not required by the task. Therefore, in addition to a possible motivation effect on change recollection, there might have been a similar effect on Phase 2 target recall more generally, including on the control trials. Regardless, the low level of recall on those trials made it difficult or impossible to observe evidence for interference. In addition, recall conditionalized on lack of awareness, even though it showed interference in Experiment 1, was too low in both experiments to reveal possible effects of meaningful connections on the degree of interference. Clearly, 72 was a greater than optimal number of trials in these experiments, and any related subsequent research should use fewer. The numerous trials that are necessary to establish power and control in experimental demonstrations, moreover, can detract from their use as analogs for real-world experience.

### Returning to application

In spite of those concerns about the application of paired-associate learning experiments, we conclude with speculations about real-world analogs for these experimental outcomes. In both applied domains that we introduced previously—misinformation correction and rumination intervention—the first-learned experiences typically dominate subsequent attempts to remember intervening changes. Original learning is the default, and the change is the exception (Bouton, 2000). Our experiments suggest that exceptions to that rule can develop through emphasis on meaningful connections between the correction or intervention and the original, problematic learning—fake news or ruminative interpretations. It is likely the case that some instruments of change seek to diminish recall of the undesirable memory by changing the subject—by connecting the shared cue with a very different kind of experience and hoping for the best in the later retrieval contest (see Miller & LaBorda, 2011). But because initial learning almost always has the edge, applications should take advantage of its inevitability by building a meaningful bridge to the change, a bridge that can be clearly noticed during correction and endure over time. The results of Experiment 2 are particularly important in that regard, because change detection can be encouraged or even explicitly incorporated in applied settings. In the real world, such external control rarely exists at the point when the correction should come to mind.

## Data Availability

Our sections on Transparency in the body of the manuscript contain addresses in OSF where data, materials, and code are available.

## Appendix

### **Descriptive Results Relevant to the Change of Instruction in Experiment 2**

**Table A1:** Mean Proportion Recalled from Data Collected Before or After the Change

Measure/Condition	Before (n = 20)	After (n = 16)
Intrusions from P1		
Connected	.22	.14
Unconnected	.39	.28
Phase-2 recall		
Connected	.40	.43
Unconnected	.18	.21
New	.20	.17
Phase-2 recall, conditional on Phase-1 recall		
Connected	.47	.48
Unconnected	.23	.22

**Note**. All comparisons of correct recall of new Phase-2 recall with correct recall in the unconnected condition did not reach significance in either instructional group, all *p* > .20. All other pairwise comparisons between conditions were statistically significant both before and after the change, *p *< .01 or .001. Counterbalancing is not preserved in this split

## Footnotes

1 We chose a medium effect size on the basis of effects sizes obtained by Hertel et al., 2023, with very similar designs and procedures.

2 One student passed on more than half of the trials; one student failed to mark any item as changed; one student failed to attempt Phase 1 recall followed a change choice; one student typed new instead of the target word in attempting recall on control trials; five students recalled fewer than two Phase 2 targets. The last group also made no distinctions on the Phase 1 image rating task.

3 One student typed cues instead of the Phase 1 targets, one recalled only one Phase 2 target (and did not speak English well), and one passed over half of the trials.

4 *F*(1,34) = 3.34, *p* = .076. No other instruction-related effect on any measure approached significance, *F* < 1.0.To alleviate possible concern, the appendix contains a table of means for the main measures, calculated according to whether the data were produced before or after the change. We note that counterbalancing was not preserved.
